# 

**Published:** 1919-07-26

**Authors:** 


### St. Thomas's Nurses in the War.

Interesting details are given in the annual report of St. Thomas's Hospital of the services rendered by the members of the staff during the war. The number of nurses sent on service abroad was 114, and the following honours were gained: C.B.E., 1; O.B.E., 1; R.R.C., 1st Class, 30; Bar to R.R.C., 2; A.R.R.C., 2nd Class, 43; Military Medal, 3; Order of St. Saviour, 1. We are glad to learn that St. Thomas's will shortly be in a position to supply private nurses. The Council of St. John's House, Bloomsbury, has transferred the Nursing Home to the Governors of St. Thomas's, and the work will be carried on under the title of St. John's and St. Thomas's House for supplying private nurses.

### THE COLLEGE OF NURSING (Limited by Guarantee).

### Award of Studentships.

Thirty-seven candidates presented themselves for examination for the second year of the College of Nursing Studentships, thirty-one of whom sat in London, three in Scotland, and three in Ireland. Of these thirty-seven, twenty-five gained over 50 per cent. of marks in the General Knowledge Paper, twenty-two in the Professional Paper, and nineteen gained over 50 per cent. of marks in both papers. Of the nineteen who gained over 50 per cent. in both papers, the records and references of nine candidates were of such a character as to lead the Selection Committee to believe they would be suitable to receive the studentships, and to qualify for such important posts as those of sister-tutors. It, however, became a difficult matter for the Council to decide who should receive the five available studentships. One candidate had to withdraw owing to circumstances over which she had no control, thus the number was reduced to eight.

On the position becoming known an anonymous friend of the College came forward offering three more studentships, to be known as London Studentships. The news that the College was thus able to send eight students to King's College next October has been received with great enthusiasm.

The Successful Nurses.

The names and the training-schools of the successful nurses for the three College, the three London, and the two Cowdray Studentships are: Miss D. M. Davis, General Hospital, Birmingham; Miss A. M. Rose, Union Infirmary, Hull; Miss E. M. F. Bowdler, Royal Southern Hospital, Liverpool; Miss E. M. Edmunds, Seamen's Hospital, Dreadnought, Greenwich; Miss D. Windley, Guy's Hospital, S.E. 1; Miss N. H. McCheane, Royal Sussex County Hospital, Brighton; Miss M. L. Lane, St. George's Hospital, London; Miss M. E. Hitch, St. Bartholomew's Hospital, E.C.

### The Cowdray Studentships.

The College is also able to announce that two studentships have been endowed by the Viscountess Cowdray, so that there will be every year two Cowdray Studentships. They are each of the value of 111 guineas, and are offered by the Viscountess Cowdray to enable trained nurses to take a course of study at the Household and Social Science Department, King's College for Women, University of London, or some other approved institution, extending over one year, to fit the holders for the appointment of sister-tutor in a nurse training-school.

Qualifications and Conditions.

Candidates for the Cowdray Studentships must be members of the College of Nursing, who have received their professional training either in Aberdeenshire or Sussex. They will be required to present themselves for a qualifying examination, which is held by the College of Nursing yearly in June. In the event of no candidate from Aberdeenshire or Sussex reaching the standard required for the course of study, the donor deputes to the College the right to allocate the studentships to nurses trained outside these counties who have passed the above qualifying examination.

This Year's Holders.

Miss McChcane has been awarded the Cowdray Studentship for this year, and as there was no student from Aberdeenshire Miss E. M. F. Bowdler, of the Royal Southern Hospital, Liverpool, was awarded the second Cowdray Studentship.

### Training-Schools in Aberdeen and Sussex.

The following hospitals and Poor-Law infirmaries in the counties of Sussex and Aberdeenshire are recognised by the College of Nursing as training-schools:—

Royal Sussex County Hospital, Brighton, Royal West Sussex Hospital, Chichester, Brighton Poor-Law Infirmary, Steyning Infirmary, Shoreham, East Sussex Hospital, Hastings, Royal Infirmary, Aberdeen, Poor House Hospital, Old Mill, Aberdeen.

LONDON CENTRE.

(7 Henrietta Street, W. 1.)

The "At Home" to the members of the London Centre at King's College Hospital, given on Tuesday, July 15, by Miss Brown, Assistant Matron, was most successful. Tea was served in the staff nurses' sitting-room, and afterwards the guests were shown over the hospital, this part of the afternoon's entertainment being not the least appreciated in view of the special interest attaching to King's College Hospital as London's newest great hospital building.

We are requested to state that the Hon. Secretary of the London Centre will be glad if all members will kindly notify her of any change in their permanent address.

EAST LANCASHIRE CENTRE.

(MANCHESTER BRANCH.)

Owing to the retirement of Miss Fletcher, R.R.C., as Local Representative of the above branch, consequent upon her leaving this area, steps were taken to appoint her successor. On a vote being taken Mrs. Burgess, M.B.E., was appointed out of nine candidates.

On Wednesday, July 9, a Garden Party was given to the members of the East Lancashire Centre (Manchester Branch) by Mrs. Burgess. M.B.E., and Mrs. Jessop Hulton at Highfield House, Worsley. About 300 invitations were sent out, and the members of the College spent a very happy and enjoyable afternoon.

### Passing Events and Topics.

### Royal College of Surgeons.

At the annual meeting of the Royal College of Surgeons, Sir Berkeley Moynihan and Sir Cuthbert Wallace were elected members of the Council.

### Chelsea Hospital for Women.

A memorial portrait of the late Earl Cadogan has been presented to the Chelsea Hospital for Women. The site of the present hospital was given by the late Earl.

### A Surgeon's Estate.

Mr. James Ashford Potts, formerly honorary surgeon at the Glasgow Royal Infirmary and the Birmingham Lying-in Charity, left £9,512.

### A Peace Cot.

A cot has been endowed in Ancoats Hospital, by gift of £500 from Mr. Aaron Lyell, of Didsbury, in memory of his mother and in commemoration of peace.

### Peace Day Gift to Incurables.

Each of the patients at the Royal Hospital and Home for Incurables, Putney, received a £1 Treasury note on the 19th inst., with the following message: "A friend who sympathises with you in your suffering sends this with the sincere wish that, though unable to take part in the public rejoicings, you may spend a peaceful and very happy day."

### Royal Institute of Public Health.

Lord Leverhulme, the President of the Royal Institute of Public Health, has issued an appeal for at least £50,000 for the establishment of an endowment fund.

### Early Preventive Treatment of Venereal Disease.

In reference to the early preventive treatment of venereal diseases suggested by the National Council, the London County Council has issued a report which is adverse to the proposal. It is stated that, with one exception, the hospital committees of all the London hospitals at which venereal clinics are in operation have definitely expressed the opinion that it would be impossible for them to introduce such treatment.

